# Cold Atmospheric Plasma in Oncology: A Review and Perspectives on Its Application in Veterinary Oncology

**DOI:** 10.3390/ani15070968

**Published:** 2025-03-27

**Authors:** André Gustavo Alves Holanda, Luiz Emanuel Campos Francelino, Carlos Eduardo Bezerra de Moura, Clodomiro Alves Junior, Julia Maria Matera, Genilson Fernandes de Queiroz

**Affiliations:** 1 Department of Surgery, School of Veterinary Medicine and Animal Science, University of São Paulo, São Paulo 05508-270, SP, Brazil; gustavoholanda.50@gmail.com (A.G.A.H.); materajm@usp.br (J.M.M.); 2 Department of Animal Sciences, Federal Rural University of the Semi-Arid, Mossoró 59625-900, RN, Brazil; luizemanuel.vet@gmail.com (L.E.C.F.); carlos.moura@ufersa.edu.br (C.E.B.d.M.); 3 Department of Natural Sciences, Mathematics and Statistics, Federal Rural University of the Semi-Arid, Mossoró 59625-900, RN, Brazil; clodomiro.jr@ufersa.edu.br

**Keywords:** comparative oncology, plasma medicine, veterinary medicine, tumors

## Abstract

Cold atmospheric plasma (CAP) is emerging as an innovative approach for cancer treatment because of its selectivity for malignant cells and absence of significant adverse effects. While modern oncological therapies face challenges such as tumor heterogeneity and treatment resistance, CAP presents itself as a low-cost and environmentally sustainable alternative. Its mechanisms of action involve reactive oxygen and nitrogen species (RONS), UV radiation, and electromagnetic fields, which induce cell death. Preclinical and clinical studies have demonstrated the efficacy of CAP, with devices such as dielectric barrier discharge (DBD) and the plasma jet developed to minimize damage to healthy cells. Some CAP devices are already approved for clinical use, showing safety and efficacy. However, the standardization of treatments remains a challenge due to the variety of devices and parameters used. Although CAP has shown promising cytotoxic effects in vitro and in animal models, especially in different cancer cell lines, further research, particularly in vivo and in veterinary medicine, is needed to optimize its clinical use and maximize its efficacy in combating cancer.

## Simple Summary

Cold atmospheric plasma (CAP) has emerged as a novel cancer therapy that acts primarily through the generation of reactive oxygen and nitrogen species (RONS). Its use presents several advantages, including selectivity for malignant cells, safety, absence of systemic adverse effects, low cost, dose-dependent effects, and activity against various tumor cell lines. Moreover, initial studies have suggested a synergistic effect with other therapies, such as surgery, radiotherapy, electrochemotherapy, and photodynamic therapy. Despite this, investigations regarding its clinical use, especially in veterinary medicine, are still nascent, necessitating further research to contribute to the establishment of this therapy.

## 1. Introduction

Cancer treatment has advanced significantly in recent decades, with the development of targeted therapies, gene editing, nanomedicine, and multimodal approaches. However, some challenges in implementing these treatments persist, such as tumor heterogeneity, resistance mechanisms, cost, difficulty of application, and adverse effects for the patient [1,2,3]. Thus, new therapeutic models that can overcome these challenges are continuously being investigated.

Cold atmospheric plasma (CAP) has gained prominence as a promising strategy in the fight against cancer and has potential in both human and veterinary medicine [4]. This technology involves the use of plasma at low temperatures and atmospheric pressure. It is generated in portable devices that are easy to handle [5] and low-cost [6] and is considered “eco-friendly” [7]. However, the available data on CAP treatment are still scarce, with no standardization of devices, application parameters, or therapeutic targets. Moreover, although several mechanisms of action contribute to the antineoplastic effects, generally, only those of chemical origin that are mediated by reactive oxygen and nitrogen species tend to be emphasized. Another challenge lies in presenting this therapy as universally effective, given that numerous factors influence the characteristics of plasma. These factors include parameters such as the applied voltage, exposure time, mode of application (direct or indirect using plasma-activated solutions), type of working gas, and flow rate in the case of plasma jets [8,9]. These variables directly affect the composition and concentration of reactive species generated by plasma, which, in turn, affect its effects on cells and tissues. The lack of standardization in these parameters often limits the relevance of findings to specific experimental conditions [5,8]. Therefore, this review seeks to consolidate concepts related to plasma therapy in oncology, particularly in the veterinary field. Thus, this paper examines CAP-generating devices and their applications, the mechanisms of action of the treatment, the main results of preclinical and clinical studies, major limitations to clinical practice and future perspectives.

## 2. Methods

This narrative literature review was conducted based on an extensive search performed between November 2021 to June 2022, in PubMed, Google Scholar, and ScienceDirect databases. Further search was conducted between July and October 2024 to update the results. The search terms included “cold atmospheric plasma”, “cancer”, “tumor”, “animal”, “human”, “in vitro”, and “in vivo”. Only articles published in English-language, peer-reviewed journals were included. Studies lacking full-text availability or those not directly addressing the application of cold atmospheric plasma in cancer research were excluded.

## 3. Mechanisms of Action of CAP

Although not yet fully understood, the main factor involved in the mechanism of biological action of its application is the production of reactive oxygen and nitrogen species (RONS), peroxynitrite (ONOO^−^), hydroxyl (OH^−^), superoxide (O_2_^−^), neutral atoms, ions, ultraviolet (UV) radiation, and the formation of an electromagnetic (EM) field resulting from the ionization of the working gas in contact with the atmosphere [5,10,11,12,13].

However, the type and proportion of generated species are directly influenced by the plasma source, so their chemical effects vary according to the generation parameters, making the standardization of treatments a challenge for clinical application. Nevertheless, the physical action of the electromagnetic field on the cells is also relevant to the outcome of the therapy. However, they act on cells only when the plasma is applied directly [9,11,14].

As a result of the interaction of electrons with the carrier gas, primary reactive species are generated, such as O_2_ and N_2_ molecules; N, O, and H atoms in the ground and excited states; and radicals. When in contact with the molecules present in the atmosphere, they form secondary reactive species known as RONS, such as singlet oxygen delta, OH^−^, NO, NO_2_, HNO_2_, HNO_3_, H_2_O_2_, and O_3_, which act as intra- or extracellular messengers or cause direct changes in the cellular structure [11,15]. Thus, the synergistic effects of reactive oxygen and nitrogen species, associated with or not associated with the physical effects of direct treatment, are responsible for inducing selective death in cancer cells both in vitro and in vivo [9].

An important factor is the half-life of these reactive species. Since they may or may not be easily degraded, they can be classified into two groups: short-lived and long-lived. The main short-lived species, such as OH^−^, superoxide anion (O2^•−^), and singlet oxygen delta (1O_2_), readily react with other atmospheric components or those present in media and tissues, making them highly relevant for direct CAP applications. Long-lived species, such as hydrogen peroxide (H_2_O_2_), nitrite (NO_2_^−^), and nitrate (NO_3_^−^), are relatively stable and persist in the environment after generation, enabling their application to the target tissue/cell hours or days after their formation, which is relevant for both direct and indirect applications [9].

### 3.1. Reactive Oxygen Species (ROS)

Atomic oxygen (O) plays an important role in the intracellular interaction of other species, as it is capable of altering cell membrane permeability, creating pores and allowing the passage of other reactive species [11]. It is also capable of reducing peripheral vascular resistance, improving blood circulation and oxygenation of ischemic tissues [16]. Another type of atomic oxygen, singlet oxygen delta, is an important short-lived reactive oxygen species that is responsible for the selective effect of CAP on cancer cells due to oxidative stress, resulting in damage to cellular genetic material [11].

Ozone (O_3_) is particularly promising for the treatment of chronic ischemia and various skin infections. It stimulates the oxidative carboxylation of pyruvate within cells, leading to increased ATP production. This process, associated with the action of atomic oxygen, helps to reduce peripheral vascular resistance and optimize blood circulation. Furthermore, ozone induces mild activation of the immune system in cells, triggering the activation of neutrophils and stimulating the synthesis of cytokines such as IL-2, TNF-α, IL-6, and IFN-γ [11,16].

Hydroxyls, which are widely used by defense cells to combat invading microorganisms, are highly reactive and can cause damage to genetic material by disrupting sugar–phosphate structures and nitrogenous bases, resulting in mutagenic processes or inducing cell death. They are also capable of inducing protein oxidation, altering protein structure, and impeding protein function, culminating in cellular dysfunction. They cause lipid peroxidation of cell membranes, altering their permeability and allowing the entry of other previously impeded molecules, inducing programmed cell death. They have a secondary immunomodulatory action as a result of cell death, promoting inflammatory stimulation with the recruitment of defense cells. As a potent reactive species, it can deplete antioxidant apparatuses such as glutathione, signaling cell death by apoptosis or inducing necrosis [11,13].

Like hydroxyl radicals, hydrogen peroxide (H_2_O_2_) is a potent reactive species that induces a significant portion of the cellular alterations caused by OH^−^ ions. However, its actions are dose-dependent. At low levels, it plays a signaling role in promoting cell growth, tissue repair, apoptosis, and angiogenesis. However, at high and unregulated levels, hydrogen peroxide can lead to cell death. An important characteristic is its ease of cellular diffusion, especially in cancer cells, due to the increased expression of aquaporins, which are integral membrane proteins that allow their entry into cells [9,11,13,17].

Superoxide radicals are important primary reactive species generated by nicotinamide adenine dinucleotide phosphate (NADPH) oxidase, a family of enzymes capable of initiating the production of reactive species, affecting biological membranes and the cellular redox balance [18]. However, they can cause direct alterations, such as suppressing the action of antioxidant enzymes; disrupting energy synthesis via mitochondrial dysfunction; causing DNA damage by oxidizing nitrogenous bases, leading to mutations and genetic instability; activating immune cells; triggering the release of proinflammatory mediators; exacerbating inflammatory responses; and directly damaging cellular components, such as lipids and proteins [11,13].

Together, reactive oxygen species can cause damage to proteins [19], induce lipid peroxidation, and alter cellular permeability, resulting in death induced by high levels of oxidative stress [17]. In response, cells increase the synthesis of antioxidant enzymes such as superoxide dismutase, glutathione peroxidase, heme oxygenase-1, and catalase to neutralize excess ROS and prevent oxidative damage [12,13,15]. Nevertheless, reactive oxygen species can activate signaling pathways involved in cell cycle arrest, apoptosis, and necrosis. These changes particularly influence cancer cells, since their antioxidant apparatus is overloaded due to their unregulated metabolic activity and constant proliferation [17,20].

Despite their detrimental capacity, ROS also serve as essential signaling molecules in the maintenance of cellular homeostasis and the regulation of physiological processes. The intricate balance between the generation of reactive oxygen species and antioxidant defense mechanisms is crucial for cellular function and adaptation to the challenges of oxidative stress. Furthermore, they play dual roles in immune responses, acting as signaling molecules and contributing to the elimination of pathogens [21,22], in addition to macrophages and neutrophils producing them endogenously to eliminate invading microorganisms through oxidative burst mechanisms [21,23].

### 3.2. Reactive Nitrogen Species (RNS)

Nitric oxide (NO) can cause apoptosis or necrosis or protect cells from death, depending on the cell type, radical concentration, and duration and specific areas of exposure. It is an important vasodilator and promoter of angiogenesis, which is necessary for maintaining blood pressure and wound healing, by stimulating the synthesis of the extracellular matrix. It acts as a signal for defense cells, mediating the inflammatory response and stimulating the immune response against pathogens. It is also an important neurotransmitter that modulates synapse formation and neuroplasticity. It also has direct action against pathogens, interfering with the metabolism of some bacteria, viruses, and parasites [11,13,15,20].

NO_x_ species, including NO and NO_2_, can adhere to lipid surfaces and interact with skin tissues, affecting pH after plasma treatment. Nitrogen dioxide (NO_2_) is a free radical that can react with other molecules in biological systems, generating new reactive species and leading to oxidative damage [15]. Together, NO and NO_2_ can induce electroporation and modifications of the biofilm membrane surface, aiding in the removal of persistent bacterial communities in chronic wounds. The interaction of NO, OH, and superoxide radicals with microbial cells can lead to oxidative damage to cellular proteins and DNA, affecting the viability of microorganisms in dermatological applications [11]. However, their direct effects on the control of neoplasms are not yet clear in the literature.

Peroxynitrite (ONOO^−^) is implicated in inflammatory processes, DNA damage, and neurodegenerative diseases because of its ability to cause nitrosative stress, demonstrating its potential as an anticancer agent that targets cancer cells and disrupts their proliferation and survival mechanisms [9,13].

Nitric acid (HNO_3_) can be generated from nitrates formed in water via plasma treatment, contributing to the acidification of lipid surfaces and skin and impacting dermatological treatments by influencing the pH of skin surfaces and lipid films, affecting the post plasma treatment recovery process [11,15].

Nitroxyl nitrogen is a less-studied reactive nitrogen species compared to nitric oxide, but it has shown potential therapeutic effects in cardiovascular diseases and ischemia–reperfusion injury since it has vasodilatory effects and acts as a cellular signaler [15]. Additionally, nitroxyl, S-nitrosothiols, and dinitrosyl iron complexes can also be found through CAP treatments. They participate in cell signaling pathways, influencing redox balance and modulating protein functions [11]. However, their effects on cancer cells have not yet been investigated in isolation.

### 3.3. Physical Effects

Upon reviewing studies on the effects of CAP on cells, it becomes evident that most approaches emphasize the role of RONS as the primary factor behind the observed biological outcomes. This focus appears to have shifted the physical action of plasma to a secondary consideration, likely owing to the lack of direct evidence available. Recently, however, interest in exploring the potential impact of physical factors, such as ultraviolet radiation, thermal radiation, and electromagnetic waves, on their interactions with cellular targets has increased.

#### 3.3.1. Electromagnetic Waves (EMs)

Plasma-generating devices can generate electromagnetic waves from 10 to 100 GHz [5,24]. Owing to the polarized nature of cell membranes, these electromagnetic pulses can interact with these structures, forming pores and altering their permeability [25].

Building upon this information, Yan et al. [14] evaluated the effects of electromagnetic waves produced by a cold plasma-generating device on a reactive species-resistant melanoma cell line (B16F10). The treatment was carried out by directing the plasma jet towards the bottom of a 96-well plate containing the cells and culture medium so that RONS would not reach them. With this method, a new type of cell death associated with CAP characterized by the rapid leakage of water through the cytoplasmic membrane was observed for the first time. The result is visible bubbling on the surface due to (1) aggregation of the cytoplasm, which creates intracellular pressure and can trigger the release of water through the cell membrane, and (2) physical damage to the cell, resulting in the formation of pores in the cell membranes through which water or cytosolic solution can be released to form bubbles. Furthermore, they reported that the cells, even after days of treatment, remained “fixed”, with no duplication or movement. Finally, it is argued that the induction of necrosis occurs more rapidly than the chemical effect of CAP since it does not depend on signaling cascades to induce programmed cell death.

#### 3.3.2. UV Radiation

UV radiation can photoreact with proteins, amino acids, and nucleic acids, resulting in nucleotide dimerization or oxidation. In excess, this leads to fibroblast senescence, erythema, premature aging, and ultimately carcinogenesis, inducing actinic keratosis, squamous cell carcinoma, and/or melanoma [25]. However, CAP is not capable of generating UV radiation at the wavelengths necessary to lead to these changes, as its wavelengths are in the range of 220 to 310 nm (UVC and UVB radiation, respectively) [26,27]. This wavelength is poorly or not absorbed by healthy skin but becomes relevant in carcinogenic induction when epidermal protection is lost [25]. Nevertheless, even for the treatment of skin lesions, exposure time to radiation is an important factor in determining the safety of CAP application. Thus, since the studied protocols do not require prolonged exposure to CAP, generally remaining in the range of 3–5 min (the time varies greatly depending on the extent of the lesion to be treated) [28], it is unlikely that the radiation generated by the plasma is sufficient to induce any degree of carcinogenesis.

Despite its carcinogenic potential, UV radiation is used as the therapeutic principle of photodynamic therapy (PDT) for the treatment of vitiligo [29], psoriasis [30], and cancer itself [31], but its potential in conjunction with cold atmospheric plasma jets has yet to be demonstrated.

The information available in the literature is scarce regarding the safety of UV radiation produced by CAP in isolation. Most studies have evaluated the synergistic effect of chemical and physical action on the target tissue/cell, making it difficult to directly relate the possible deleterious effects to the ultraviolet radiation produced. To date, the safety of this treatment has been widely defended for short-term therapies (approximately 3 continuous minutes) [26,27,28,32].

#### 3.3.3. Thermal Radiation

The safety of plasma treatment has facilitated the expansion of in vivo studies. Saadati et al. (2018) [33] evaluated the skin temperature of mice via thermography after 6 min of exposure to CAP and reported an increase of only 1 ±  0.3 °C, which was insufficient to cause thermal damage. Similarly, in a previous clinical case, we reported a slight increase in skin temperature from 35 to 35.7 °C after CAP treatment [32]. Furthermore, according to the available literature, the thermal radiation produced during CAP generation does not appear to significantly impact the antitumor effects. For devices designed for biomedical applications, temperatures typically remain below 40 °C [34], as also demonstrated in our previous study [32]. However, CAP treatment has the potential to increase the sensitivity of neoplastic cells to treatments based on thermal radiation, such as photothermal therapy (PTT). For instance, Qin et al. (2022) [35] demonstrated that CAP, when combined with gold nanostars as photothermal agents, selectively increased the susceptibility of cancer cells to PTT. Compared with PTT alone, this combination allowed for effective tumor suppression with reduced side effects. Nevertheless, whether the thermal radiation produced by CAP alone exerts a significant antineoplastic effect remains uncertain.

**Figure 1:**
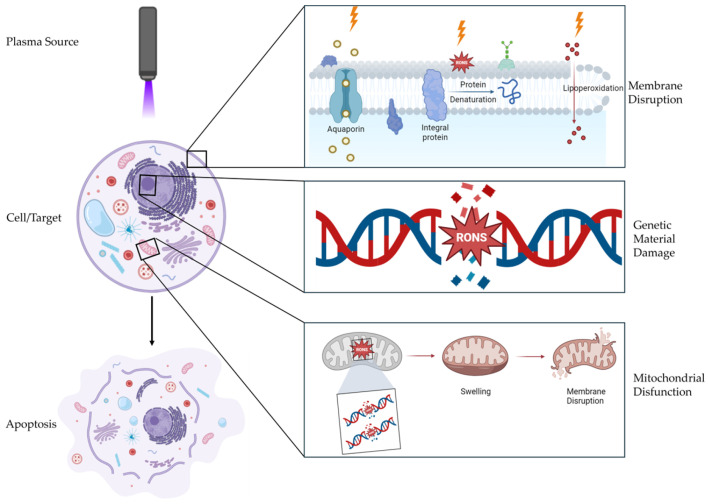
Mechanistic overview of plasma-induced apoptosis in target cells. Reactive oxygen and nitrogen species (RONS) generated by plasma cause membrane disruption, genetic material damage, and mitochondrial dysfunction, ultimately leading to selective apoptotic cell death. (Source: Figure created by the authors). Created in BioRender.com (accessed on 25 January 2025).

A summary of the main effects of cold atmospheric plasma treatment on neoplastic cells is shown in Figure 1.

## 4. CAP Devices for Clinical Use

CAP devices used in clinical practice are designed to generate a plasma plume capable of avoiding damage to healthy cells and are not associated with significant side effects [36]. Three main approaches have been used to generate CAPs: (1) dielectric barrier discharge (DBD), which produces direct plasma discharges; (2) plasma jets, which generate indirect discharges; and (3) hybrid devices, which integrate both methods [37]. The characteristics, applications, and limitations of these approaches are summarized in Table 1.

In DBD devices, where the discharge is direct, plasma is ignited in the gap between an isolated (dielectric) high-voltage electrode and the tissue or living cells may serve as a counter electrode, actively participating in the discharge process without the need for carrier gasses [38]. They provide more intense, adaptable, and controlled plasma discharge, cover large areas without requiring additional gas supply equipment, and use atmospheric air as a working gas, although an artificial atmosphere can also be utilized. The main limitation of these methods is the need for a constant distance, which requires a smooth and flat surface [39,40].

The plasma jet is classified as indirect, as the electrodes responsible for plasma generation are located in the device itself, and the active plasma species are transported to the target (i.e., cells, tissue or tumor) by a gas flow, such as helium, argon, or oxygen [38]. During transport to the target, there will be a reduction/extinction of charged particles, UV radiation, and short-lived species. In this device, plasma is typically released in a ring-shaped structure with the gas flowing through the central channel. This flow produces the “jet” that gives these devices their name [41]. The main advantage of the plasma jet is its ease of handling and suitability for treating different surfaces. The main limitation is the loss of a significant portion of plasma through the nozzle or orifices, resulting in less efficient generation compared with DBD devices [42].

Hybrid devices share the benefits of direct and indirect sources but are currently applied only at the experimental level [36,39]. These devices combine microdischarges on a grounded mesh electrode. The discharge produced is uniform, with no effect on the object between the two electrodes, and the device is relatively easy to control. However, hybrid devices have a slightly greater susceptibility to component wear and subsequent deterioration. This phenomenon is especially noticeable in humid environments and follows direct contact with treated cells and tissues [39].

Currently, there are plasma-generating devices approved and marketed for clinical use, such as the KINPen MED plasma jet (INP Greifswald/neoplas tools GmbH, Greifswald, Germany) [43], PlasmaDerm VU-2010–DBD (CINOGY Technologies GmbH, Duderstadt, Germany) [44], and the SteriPlas plasma jet (Adtec Ltd., London, UK) [45]. Experimentally, these devices have been developed and adapted. They are safe and easy to operate, with a low manufacturing cost and satisfactory clinical results [32,46,47].

**Table 1:** Cold atmospheric plasma generation mode and characteristics based on device type.

Device Type	Generation Mechanism	Key Characteristics	Common Clinical Applications	Limitations
Dielectric Barrier Discharge (DBD)	- Generates direct plasma discharge between two electrodes separated by a dielectric barrier [37].	- Cover larger areas more easily [40]. - Does not require additional gas supply equipment [40].	- Treatment of superficial acute or chronic wounds, skin disinfection, and tissue regeneration [44,48].	- Less effective on irregular surfaces [39].
Plasma Jets	- Generates indirect plasma discharge by expelling ionized gas in a directed jet [41].	- Produces plasma at a distance from the generating electrode [38]. - Greater adaptability treating for irregular surfaces [32,42].	- Treatment of superficial acute or chronic wounds, skin disinfection, and tissue regeneration [49,50]. - Suitable for therapies for delicate tissues, such as mucous membranes [51].	- Loss of a significant portion of plasma through the nozzle other openings [42].
Hybrid Devices	- Combines microdischarges on a grounded mesh electrode [36].	- Integrates advantages of both DBD and plasma jets [36]. - Produces a uniform discharge [39]. - Device is relatively easy to control [39]	- Currently applied only at the experimental level [39].	- Increased susceptibility to component wear and subsequent deterioration [39].

## 5. Applicability of the CAP

Knowledge regarding the applicability of CAP in cancer therapy is limited to in vitro studies, animal models, and a few clinical studies [32,52]. On this basis, several advantages have been proposed for its use, including (1) the ability to selectively destroy malignant cells while preserving normal cells in the body, minimizing the local toxicity of treatment [53,54]; (2) the absence of systemic adverse effects [28,32]; (3) dose-dependent antineoplastic effects [55]; and (4) synergistic action with other therapeutic modalities (e.g., surgery, radiotherapy, electrochemotherapy) [41,56,57]. Surgery can reduce the challenge of plasma penetrability in proliferative tumors, and CAP can help prevent tumor recurrence after surgical excision by destroying residual neoplastic cells [58].

CAP can be applied to affected tissues via direct exposure [59] or indirect exposure [60]. Direct exposure, despite the advantage of exposing the target to the greatest number of species produced by plasma, has the drawback, when used in cancer treatment, of inducing patient discomfort owing to the accumulation of electrical charges, in addition to the difficulty of molding to the target area [61,62].

On the other hand, indirect exposure through plasma-activated solutions (PASs), obtained by treating a sterile solution (e.g., saline or Ringer’s lactate) with plasma, or plasma-activated medium (PAM), derived from exposing a culture medium to plasma and subsequently injecting it in vivo, can help mitigate discomfort and improve accessibility to hard-to-reach tumors [63]. Hypothetically, the injection of PAS or PAM may make it possible to approach more proliferative, subcutaneous, or intraperitoneal tumors [64,65]. The administration of indirect plasma to subcutaneous tumors is relatively easy, as there is typically a single visible tumor mass, enabling precise and effective injections. However, in internal tumors disseminated throughout the peritoneal cavity, visibility and reach may be impaired [66].

Regarding the mechanism of action, it remains uncertain whether the anticancer effects of direct and indirect treatments are equivalent. In direct treatment, the treated tissue is exposed to both the physical (e.g., high electric fields, ultraviolet radiation, and thermal radiation) and chemical components (e.g., free radicals and neutral molecules) of CAP. In contrast, indirect treatment eliminates all the physical aspects of CAP, retaining primarily long-lived RONS (e.g., H_2_O_2_, NO_2_^−^, NO_3_^−^, and ONOO^−^) [9,13].

## 6. Preclinical Trials

### 6.1. In Vitro Trials

The first series of studies to evaluate the effects of CAP on cancer cells date back to 2010, when Kim et al. [67], Zirnheld et al. [68], and Georgescu and Lupu [69] presented results indicative of plasma selectivity toward cancer cells, especially melanoma cells.

In 2011, Keidar et al. [70] published the first article with more robust results and better-explained mechanisms regarding the application of CAP in neoplasms in vitro and in vivo, once again demonstrating its selective effect, now in the SW900 (human lung cancer) and B16-F10 (murine melanoma) cell lines. It has been demonstrated that cold plasma induces apoptosis and reduces cell migration, highlighting its application for cancer treatment by localizing tissue damage and limiting metastasis. Furthermore, the selective action of cold plasma on different cell types suggests its ability to eradicate cancer cells while preserving healthy cells. The primary reason for this selectivity is increased basal ROS levels in cancer cells due to their robust metabolic activity. As a result, when additional ROS stress is induced through CAP treatment, oxidative stress more easily induces apoptosis by disrupting membrane components, proteins, lipids, and genetic material in cancer cells than in normal cells [65]. Additionally, cancer cells tend to express more aquaporins on their cytoplasmic membranes, which may facilitate faster H_2_O_2_ uptake in cancer cells (Figure 1) than in normal cells. Furthermore, CAP has demonstrated a dose-dependent antineoplastic effect [71,72,73] and selectivity for malignant cells [32,73,74]. The primary effects on mutated cells include apoptosis, growth inhibition, cytoskeletal disruption, cell cycle arrest, genetic damage, lipoperoxidation, and immunogenic cell death, which are driven primarily by the accumulation of reactive oxygen and nitrogen species [5,65]. Two general effects related to the vulnerability of cancer cells to CAP treatment have been observed. First, cancer cells that express the p53 gene mutation are more resistant to CAP treatment than are cells that do not express this mutation [75]; second, cancer cells with higher proliferation rates are more sensitive to CAP treatment than are cells with lower proliferation rates [76]. Mechanistically, CAP treatment causes the apoptosis of cancer cells through a selective rise in intracellular ROS and corresponding ROS-based death pathways. The apoptosis of CAP-treated cancer cells may be due to severe DNA damage, mitochondrial damage, cytoplasmic membrane damage, or a weakened intracellular antioxidant system [5]. Consequently, CAP treatment more effectively eliminates cancer cells while sparing normal cells [77].

Until 2018, 94.7% of the research involving CAP and cancer was conducted in vitro, with minimal adverse effects reported and no signs of treatment resistance mechanisms [62]. Other studies have subsequently demonstrated the antineoplastic effects of CAP against various cell lines, including cutaneous squamous cell carcinoma [78], lung cancer [79], osteosarcoma [80], cholangiocarcinoma [81], pancreatic cancer [82], colon cancer [55], prostate cancer [53], lymphoma [83], head and neck carcinoma [32], breast cancer [37], glioblastoma [71], and ovarian cancer [84]. To our knowledge, the only study conducted with a pet animal cell line was canine osteosarcoma, which showed promising antineoplastic effects for veterinary medicine, including apoptosis induction and a reduction in migration and cellular invasion activities [85].

The conclusions obtained from in vitro studies cannot be directly used to predict the performance of in vivo studies. The relatively dry skin barrier between the plasma and the tissues or cancer cells under the skin is different from the experimental conditions produced in vitro. The in vitro environment presents a medium layer that facilitates the transition of some short-lived reactive species in the gas phase to long-lived reactive species in the liquid phase. In addition, both long-lived and short-lived reactive species undergo complex reactions at this gas/liquid interface. Therefore, in vivo studies play a fundamental role in the development of plasma medicine [86].

### 6.2. In Vivo Trials

The anticancer effects of plasma have been verified in animal models of a variety of neoplasms, including cutaneous melanoma [55,87], cutaneous squamous cell carcinoma [78], glioblastoma [71,88], cholangiocarcinoma [81], lymphoma [83], breast cancer [89,90], pancreatic cancer [91,92], ovarian cancer [93,94], colon cancer [55,95,96,97], oral carcinoma [98], head and neck cancer [99], gastric cancer [64], and lung cancer [98,100].

Preclinical in vivo studies with CAP have demonstrated several positive effects in cancer treatment, whether by direct or indirect exposure, including dose-dependent antineoplastic effects [55], tumor volume reduction [55,96], tumor growth reduction [87,89,93,97], prolonged survival [33,89,92,94], increased expression of apoptosis markers [55,89,91], reduced expression of cell proliferation markers [91,98,99], reduced occurrence of intraperitoneal metastases [64], absence of systemic adverse effects [28,64,92,98], potentiation of chemotherapeutic drugs [87,88], induction of immunogenic cell death [89,94,95], and occurrence of the abscopal effect [89,97].

The use of intraperitoneally administered PAS has shown promising results in models of intra-abdominal tumors. Takeda et al. (2017) [64] utilized a mouse model established via intraperitoneal injection of gastric cancer cells (GCIY-EGFP cells) to assess the efficacy of plasma-activated medium (PAM) in inhibiting peritoneal metastasis formation. The results indicated that the median number of peritoneal metastatic nodules larger than 1 mm was 10 (range 7–15) in the control group and 0 (range 0–6) in the PAM group. The percentage of peritoneal metastasis formation was 100% (5/5) in the control group and 40% (2/5) in the PAM group, with no adverse events observed. Nakamura et al. (2021) [94] investigated intraperitoneal lavage via plasma-activated lactate Ringer’s solution (PAL) in murine ovarian cancer (ES2) cells. The authors observed the recruitment of M1-type macrophages to the tumor, indicating that PAL might trigger an antitumor immune response. Furthermore, intraperitoneal PAL administration significantly inhibited the progression of ovarian cancer in the abdomen, increasing the overall survival rate.

Plasma has demonstrated a synergistic effect with other treatment modalities. One study applied CAP to residual tumors (4T1 breast tumor/B16F10 melanoma) immediately after surgical resection. Compared with untreated mice (surgery-only group), CAP-treated mice presented significantly better control of tumor regrowth and prolonged survival. Furthermore, an increase in calreticulin levels was observed in residual tumor tissues after CAP treatment, suggesting the emergence of an anticancer immune response [101]. Another study evaluated the combination of CAP with radiotherapy in the treatment of hepatoblastoma (HepG2 cells) in mice. The treatment resulted in a reduction in tumor volume of 19.7% for the group treated with CAP, 35.4% for the radiotherapy group, and 53.7% for the group that received both treatments. Statistical analysis demonstrated a significant difference among the three groups, reinforcing the potential for this novel therapeutic strategy in malignant tumor control [102].

The enhancement of the antitumor effects of electrochemotherapy in a melanoma model (B16–F10 cells) has also been reported. The results revealed that the untreated mice survived an average of 28.5 days. Electrochemotherapy (ECT) combined with a plasma jet (ECJ) proved to be the most effective, extending survival by an additional 24 days compared with that of the controls, followed by ECT alone, which resulted in a significant increase of 19 additional days. ECT combined with dielectric barrier discharge plasma (DBD) led to a nonsignificant increase of 7.6 additional days. Neither DBD nor the plasma jet alone significantly impacted survival, with the plasma jet increasing survival by only 3.6 days [56]. Plasma has also been shown to enhance the antineoplastic effect of chemotherapeutics. A study investigating the combination of CAP and doxorubicin for the treatment of breast cancer (4T1 cells) in mice demonstrated superior tumor inhibition to that of doxorubicin alone while also reducing chemotherapy-associated liver damage and nephrotoxicity [90]. A summary of preclinical in vivo studies conducted in mice is presented in Table 2.

## 7. Clinical Trials

Plasma therapy has shown good results for human patients with actinic keratosis, a preneoplastic lesion that precedes cutaneous squamous cell carcinoma [56,103,104,105]. In the study by Daeschlein et al. [56], CAP was effective in treating a scalp lesion refractory to treatment with photodynamic therapy, ablative lasers, and cryosurgery. On the other hand, Friedman et al. [103] reported a complete resolution of 53% (9/17) of lesions after a single treatment of 1–2 min. In parallel, other authors obtained a clinical response in 55% of the evaluated lesions and a complete resolution of 23% with the administration of CAP twice a week for 2 min over a month in a large number of samples [104].

Clinical trials have been conducted to evaluate plasma treatment in humans with locally advanced head and neck squamous cell carcinoma undergoing palliative treatment. The observed benefits include a reduction in bacterial contamination, pain control, and improvement in patients’ quality of life [106,107,108]. One study reported a clinical response in approximately one-third (4/12) of patients [107,108]. Similarly, another study reported a partial response in 33% (2/6) of individuals, with an 80% reduction in the tumor area in one of the individuals [108]. The treatment is considered safe, and the adverse effects are mild and local, including pain, bleeding, edema, sialorrhea, and necrosis [106].

A phase I clinical trial was conducted with 20 human patients with stage IV or recurrent tumors of various histological origins. The patients underwent treatment, which consisted of a combination of surgery and CAP administration in the surgical bed. The therapy demonstrated safety and potential for preventing cancer recurrence, even in patients with compromised margins [58].

To our knowledge, the only veterinary clinical case was conducted on a feline with three cutaneous squamous cell carcinomas. The therapy demonstrated clinical benefit, with one patient in complete remission, one in partial remission, and one with stable disease for another lesion. Complete remission was confirmed histologically. The two remaining lesions presented increased expression of caspase-3 and TNF-alpha, suggesting the induction of cancer cell apoptosis. Adverse effects were limited to erythema and crusting in the treated areas [32].

The protocols used for the treatment of tumors in humans [106,107,108] and in veterinary medicine [19] are similar, consisting of treatment cycles. Each cycle consisted of 3 weekly plasma administrations, followed by one week without exposure. The tumor area was continuously scanned for 1 min/cm^2^ at a distance of 8 mm. The voltage discharge ranged from 2 kV to 3 kV at a frequency of 1 MHz and was modulated at 2.5 kHz with a plasma duty cycle of 1:1. The working gas can be argon or helium at a flow rate of 2 L/min to 6 L/min.

## 8. Limitations and Perspectives

The clinical use of plasma in oncology is promising, but some limitations must be considered. Direct plasma exposure has a limited penetration depth for RONS, restricting its application to superficial skin tumors [62]. However, combining plasma treatment with surgery during the intraoperative period enables access to deeper tissues, helping to control compromised margins and preventing tumor recurrence, but increasing the invasiveness of the procedure. Furthermore, prior surgical excision can reduce the challenge of plasma penetrability. In addition, its selectivity for malignant cells makes the treatment safer [58].

Alternatively, indirect treatment via plasma-activated medium (PAM) injection may allow access to subcutaneous tissues or body cavities [64,65]. However, it remains an open question whether the mechanisms of action of direct and indirect treatments are the same in terms of their anticancer effects. In direct treatment, the target tissue is exposed to both the physical (e.g., high electric fields, ultraviolet radiation) and chemical components (e.g., free radicals, neutral molecules) of CAP. In contrast, indirect treatment eliminates all physical aspects of CAP, leaving primarily long-lived RONS (e.g., H_2_O_2_, NO_2_^−^, NO_3_^−^, and ONOO^−^) [9,13].

CAP can also exert a synergistic effect with chemotherapy, enhancing the cytotoxic effects of drugs, promoting the process of cell death via apoptosis and mitigating the phenomenon of chemoresistance by downregulating drug resistance genes [109,110]. In addition, CAP can significantly increase the antineoplastic effects of radiotherapy [57,111], photodynamic therapy [111], and electrochemotherapy [56], suggesting the potential for sensitizing cancer cells to treatment. The use of CAP can also induce a phenomenon known as plasmaporation, which involves the generation of pores in the cellular membranes [112], facilitating the influx of chemotherapeutic agents and potentiating electroporation [112,113].

Another perspective for the clinical use of CAP is the control of local infection and pain relief, which can be useful for patients with ulcerated, infected, and painful tumors in palliative care [108]. Nonetheless, although CAP is considered safe and well tolerated, potential adverse effects include crusting, erythema, edema, pain, bleeding, burning, and necrosis [28,32,106].

Furthermore, one of the major challenges in integrating cold plasma into clinical practice is the lack of standardization in experimental setups. As of today, there are many variations in plasma-generating devices, direct and/or indirect application, treatment protocols, and exposure parameters, which hinders reproducibility and make cross-study comparisons difficult [9,114]. This way, the next critical step for clinical and surgical applications of CAP involves a deeper understanding of its generational parameters and their influence on plasma interactions with the cells and tissues. This way, therapeutic protocols can be established more objectively, since even if increasing the parameters of intensity and time of cell exposure to CAP can directly influence its antineoplastic effects, it can also increase the likelihood of adverse effects [115].

## 9. Conclusions

CAP is a promising therapy with versatility in the production of different entities, including electromagnetic fields, UV radiation, charged particles, and short- and long-lived species, which can inhibit proliferation, reducing the viability of cancer cells of various origins. CAP has potential for clinical use in human and veterinary oncology because of its antineoplastic effects, selectivity for malignant cells, and absence of systemic adverse events. Its versatility allows its combination with other established therapies, such as surgery, chemotherapy, radiotherapy, photodynamic therapy, and electrochemotherapy. Future studies are needed to understand the role of CAP in the treatment of people and animals with cancer, especially in veterinary medicine, which has a limited number of trials, both in vitro and in vivo.

## Figures and Tables

**Table 2:** List of preclinical in vivo studies with CAP against tumor models in mice detailing the device utilized, the experimental parameters for the treatment, and the main results achieved.

Tumor Model	Plasma Device	Exposure (Mode/Time)	Results
Melanoma (B16F10 cells) [56]	kINPen 09^®^ plasma jet (argon gas, flow rate ~ 8 L/min) and DBD plasma, voltage (14 kV), pulse repetition rate (100-400 Hz), electric powers dissipated in the gas discharge (167–237 mW)	Direct exposure/exposure for 2 × 3 min with DBD (30 s pause after each 3 min treatment) and 5 min with plasma jet	Synergism with electrochemotherapy, leading to prolonged survival
Pancreatic cancer (6606PDA cells) [92]	kINPen MED^®^ plasma jet/argon gas	Indirect exposure/intraperitoneal injection/1 mL of plasma-activated medium (PAM) for 10 min	Tumor growth reduction; increase in median survival and apoptosis; decrease in tumor proliferation; treatment considered safe
Gastric cancer (GCIY-EGFP cells) [64]	Plasma jet/argon gas/10 kV, powered by a 60 Hz commercial power supply	Indirect exposure—intraperitoneal injection/6 mL of plasma-activated medium (PAM) for 5 min	Tumor growth reduction; increase in median survival and apoptosis; decrease in tumor proliferation; treatment considered safe
Hepatoblastoma (HepG2 cells) [102]	Plasma jet/argon and oxygen gas/output voltage (3 kV), current (40 mA), average power 12 W	Direct exposure for 20 s	Tumor volume regression and increased apoptosis, particularly in combination with radiotherapy; treatment considered safe
Cutaneous squamous cell carcinoma (UV induced skin cancer) [78]	kINPen^®^ plasma jet/argon gas	Direct exposure for 3 min	Reduced progression of UVB-induced SCC-like lesions; decreased cell proliferation
Cholangiocarcinoma (EGI-1 cells) [81]	Plasma jet/helium gas (flow rate 1 L/min), amplitude (9 kV), duty cycle (14%), repetition frequency (30 kHz)	Direct exposure for 1 min	Tumor size reduction and decreased growth rate; induction of DNA damage and tumor cell apoptosis
Ovarian cancer (ES2 cells) [94]	Plasma jet/argon and oxygen gas (flow rate 2 L/min), 10 kV, powered by a 60 mA 60 Hz commercial power supply	Indirect exposure—intraperitoneal injection/10 mL of/Ringer’s solution activated for 5 min	Inhibited tumor progression; improved overall survival; activated immune response
Breast cancer (4T1 cells) [89]	Plasma jet/helium gas/frequency (20 kHz), total input power (1 W)	Direct exposure for 300 s	Tumor growth and weight reduction; abscopal effect; increased survival and apoptosis; induction of immunogenic cell death
Melanoma (B16F10 cells) and breast cancer (4T1 cells) [101]	Portable ambient air-fed CAP (aCAP)/voltage (6 V) and flow 16.8 L/min	Direct exposure for 1, 2, 3 and 4 min	Synergism with surgery, leading to tumor growth inhibition, prolonged survival, and induction of cancer immunogenic cell death
Melanoma (A375 cells), oral squamous cell carcinoma (Tca-8113) and lung cancer (A549) [98]	DBD plasma/voltage applied to the electrodes was sinusoidal at 15 kHz with a peak-to-peak voltage of 6 kV; the total power was 40 w	Indirect exposure—intratumoral injection/5 mL of saline activated for 10 min	Inhibition of tumor growth and cell proliferation; treatment considered safe
Colorectal carcinoma (CT26 cells) [97]	DBD plasma/High voltage pulses (25 kV) with 20, 60, or 90 ns pulse width at a repetition rate of 100 pulses/s.	Direct exposure for 10 min	Tumor growth limitation; abscopal effect
Breast cancer (4T1 cells) [90]	Plasma jet/Argon gas (flow 2 L/min) or helium (flow 4 L/min) and oxygen (flow 0.2 L/min) gas, voltage (20 kV), frequency (18 kHz)	Indirect exposure/intratumoral injection/5 mL of medium activated for 3 min	Inhibition of tumor growth, particularly in combination with chemotherapy (doxorubicin); reduced doxorubicin-induced liver and kidney toxicity
Melanoma (B16F10 cells) and colon cancer (MC38 cells) [55]	MediPL^®^ plasma torch system/argon gas (flow 2 L/min)	Direct exposure/exposure for 2, 5, or 15 min	Reduction in tumor volume and weight; decreased tumor proliferation; dose-dependent apoptosis.
Head and neck cancer (Fadu and SCC7 cells) [99]	Piezobrush^®^ PZ2/piezoelectric direct discharge technology	Direct exposure for 1 min	Reduced tumor growth and weight; prolonged survival; synergistic effect with immune checkpoint blockade (PD-L1) and chemotherapy (cisplatin).
Glioblastoma (U-87 MG cells) [71]	Plasma jet/helium or argon gas (flow 2 L/min)/frequency (20 kHz), voltage (4.5 kV)	Direct and indirect exposure/variable exposure time, based on IC50 values obtained from in vitro results	Reduction in tumor size; increased survival rate; improved general motor function.

## Data Availability

No new data were created or analyzed in this study. Data sharing is not applicable to this article.
